# Self-stigmatization of high-school students seeking professional psychological help: the chain-mediating effect of perceived social support and optimism

**DOI:** 10.3389/fpsyt.2023.1289511

**Published:** 2023-11-13

**Authors:** Nanyin Bu, Zuoshan Li, Jiarui Jiang, Xin Chen, Ziying Li, Yujuan Xiao, Xueyan Wang, Tianyi Zhao

**Affiliations:** ^1^Key Laboratory of Applied Psychology, Chongqing Normal University, Chongqing, China; ^2^School of Teacher Education, Chongqing Normal University, Chongqing, China

**Keywords:** high-school students, help-seeking self-stigmatization, professional help-seeking attitudes, perceived social support, optimism

## Abstract

**Background:**

Research has shown that stigmatization of professional psychological help-seeking is an important factor influencing attitudes toward seeking professional psychological help (ATSPPH). However, how perceived social support (PSS) and optimism have a mediating role is not clear.

**Objective:**

Examine the associations between ATSPPH, self-stigmatization of seeking help, PSS, and optimism in a cohort of Chinese high-school students (HSSs).

**Methods:**

An offline survey was conducted in three high schools in Chongqing (China) from 20 February to 20 May 2023. Participants were HSSs recruited through their teachers. A total of 2,159 HSSs completed a survey on demographic information as well as the Self-Stigmatization of Seeking Help (SSOSH) score, ATSPPH, Perceived Social Support Scale (PASS), and Life Orientation Test (LOT). Mediation analyses were conducted using the “Process” macro in SPSS 26.0 to estimate the direct and indirect effects of self-stigmatization of seeking psychological help on ATSPPH.

**Results:**

Self-stigmatization of seeking psychological help was significantly and negatively related to ATSPPH among HSSs. Self-stigmatization of psychological help-seeking influenced ATSPPH through three pathways: (a) separate mediating effect of PSS (effect = −0.029); (b) separate mediating effect of optimism (effect = −0.069); (c) chain-mediating effect of PSS and optimism (effect = −0.017). These data suggested that self-stigmatization of psychological help-seeking could influence ATSPPH directly and indirectly through PSS and optimism.

**Conclusion:**

PSS and optimism mediated the relationship between self-stigmatization of seeking help and ATSPPH. Improving the ability of HSSs to perceive social support and cultivating optimism could help improve the self-stigmatization of help-seeking and promote a positive attitude toward professional help-seeking.

## 1. Introduction

Adolescence is the transition period from childhood to adulthood. This change has a dramatic effect upon physiology and psychology. Some studies have suggested that these changes may lead to adjustment problems such as depression (1), anxiety (2), mood disorders (3, 4), substance-use disorders (5), and suicidal ideation (6).

High-school students (HSSs) are in the early stages of adolescence. The dramatic changes wrought by adolescence and the pressure of academic competition can lead to psychological distress, as well as the need to seek professional services for psychological help. However, due to the stigmatization associated with professional counseling, the demand for such professional services is not strong. A survey by Chen et al. (7) showed that the prevalence of detection of mental health (MH) concerns among Chinese adolescents was 26.3%. A large-scale, cross-sectional epidemiology study showed that the prevalence of depression and anxiety symptoms among Chinese secondary-school students was 43.7 and 37.4%, respectively. In addition, the comorbidity prevalence of depression and anxiety symptoms among students was 31.3% (8). In China, the prevalence of depressive symptoms during the coronavirus disease-2019 epidemic was higher in adolescents than in adults (9). The uncertainty of the incubation period of severe acute respiratory syndrome-coronavirus-2 and its possible asymptomatic transmission cause additional fear and anxiety. This phenomenon can lead to significant psychological stress in a population, with a corresponding increase in the demand for mental-health services (10). In China, several mental-health institutions, mental-health education and counseling centers, and mental-health associations for college students have adopted online mental-health services to provide psychological counseling as well as crisis-intervention services through hotlines and mobile application platforms (11). However, not many adolescents took the initiative to seek professional psychological help (PPH) if they had psychological concerns (12).

Fischer and Turner (13) defined attitudes toward seeking professional psychological help (ATSPPH) as an individual’s tendency to seek or refuse professional psychological assistance during a personal crisis or chronic psychological discomfort. That is, the level of tendency of an individual to actively seek help from professionals/institutions if encountering psychological, behavioral, or emotional problems that are difficult to resolve (14). A report by the National Mental Health Development in China (2019–2020) showed MH concerns to be more prevalent in youth. Only 30.8% of the 29,045 adolescents surveyed indicated that they would seek PPH if they had psychological concerns (15). A MH survey of 1,399 adolescents (14–15 years) in China, Japan, and South Korea showed that some students indicated that they would ask their peers and teachers for advice on study-related problems and difficulties with peers and family relationships. However, they would be less willing to seek professional counseling and help if they had MH concerns (16). Those data suggest that, in the context of Eastern culture, different standards for social interactions, family values, views on psychological concerns, and other factors have different degrees of influence on the attitude of adolescents toward seeking psychological help. Those data also suggested that low degree of social and cultural stigmatization of mental concerns and psychological help-seeking behaviors was conducive to the formation of a positive attitude toward psychological help-seeking in an individual (17).

HSSs are at a stage of huge physiological and psychological changes. These changes are manifested in an uneven development of individual factors (psychological, cognitive, and physiological) during adolescence but adolescents also experience interactions of complex environmental factors such as school, peers, family, and community (18). These factors can lead to the development of psychological concerns, and even extreme behaviors, such as harming others or suicidal ideation (19). Also, the stigmatization of MH concerns and the stigmatization of seeking PPH may be important factors that prevent individuals from solving their problems by seeking professional psychological help (20). Seeking psychological help is a self-relevant process, so individuals who feel stigmatized by seeking psychological help believe that seeking help is not conducive to maintaining a positive image of themselves (21). Even if they have psychological concerns, they are reluctant to seek PPH to avoid the social stigmatization and negative evaluations from others associated with the act of seeking help (22). Therefore, exploring the relationship between the self-stigmatization of HSSs toward seeking PPH and their ATSPPH is very important for: (i) overcoming the stigmatization effect on seeking professional psychological counseling; (ii) helping students with psychological concerns seek professional psychological counseling to solve their problems.

From the perspective of social construction, Crocker pointed out that the essence of stigmatization is “devaluation of social identity” (23), and that stigmatization is the result of the interaction of labeling, stereotyping, exclusion, loss of status, and discrimination (24). Corrigan divided stigmatization into “public stigmatization” and “self-stigmatization” (25). Specifically, public stigmatization is the shape of self-stigmatization, and the latter is the internalization of norms by individuals affected by public stigmatization. Self-stigmatization is a more important factor than public stigmatization because it prevents individuals from seeking psychological help (26). Stigmatization resulting from associated psychological concerns and mental disorders with negative evaluations is a barrier to individuals seeking PPH (25, 27). Symptoms of many serious mental disorders, such as inappropriate emotions and bizarre behaviors, produce stigmatized responses (28, 29). Alluhaib et al. showed that public attitudes and stigmatization toward MH disorders discourage individuals from seeking PPH (30), and that self-stigmatization toward psychological concerns (e.g., depression) is significantly negatively correlated with the attitude toward seeking professional help (12, 31). Individuals may believe that acknowledging the need for help implies their own weakness and incompetence. This underlying stigmatization may influence their attitudes and actions toward seeking psychological help (32). Self-evaluations and social expectations related to seeking psychological help can influence the self-stigmatization of individuals seeking PPH, which further affects their willingness to seek help. Accordingly, Hypothesis #1 can be proposed: self-stigmatization of seeking psychological help is a negative predictor of ATSPPH.

Perceived social support (PSS) is an emotional experience in which individuals feel understood, supported, and respected in society, and have a level of satisfaction of their lives (33). This is an important coping resource for individuals to adapt to society (34). Studies have shown that the social support perceived by HSSs is closely related to the attitude toward seeking psychological help. For example, support from teachers can increase an individual’s willingness to seek psychological help significantly (35). Support from friends, classmates, and family members also affects an individual’s attitude toward seeking psychological help (36, 37). The higher the PSS level, the more positive is the attitude toward seeking help (38). An individual’s level of PSS significantly predicts his/her attitude toward seeking psychological help and promotes individuals to seek professional help; the higher the PSS level, the more positive is the attitude toward seeking psychological help, and the higher the likelihood of seeking psychological help (39). In particular, the support of family and friends plays an important part in this process, and prompts help-seeking behavior by an individual (40). Those data show that understanding social support affects an individual’s attitude toward seeking psychological help. The results of a study on the stigmatization experience of 36 patients suffering from schizophrenia showed that internalized stigmatization was related to social support (41). Schizophrenia is hereditary, but several factors can exacerbate this affliction. For example, the labeling and stigmatization of “mental disorder” results in negative social reactions that can exacerbate the course of the disorder for the sufferer (42, 43). In the context of a mental disorder, it is often assumed that talking about traumatic experiences exacerbates distress (44). Due to the stress sensitivity of mental-disorder symptoms (45), interventions involving distressing content may exacerbate symptoms and increase the risk of relapse (46).

Research has shown that self-stigmatization of psychological help-seeking is significantly and negatively related to PSS (47). Family, friends, service providers, and employers are important sources of social support. Hence, experiences and perceptions of negative reactions can adversely affect an individual’s life. The inability of an individual to receive support from these sources can expose the individual to discrimination and treatment (48) which, in turn, leads to feelings of rejection, marginalization, and neglect (49, 50), thereby enhancing self-stigmatization (51). Thus, individuals with lower self-stigmatization for seeking psychological help tend to have lower levels of PSS (52).

Comprehensive cognitive modeling of self-stigmatization suggests that social support is one of the protective factors of self-stigmatization, and that the power of social support can ameliorate self-stigmatization and stress due to shame (53, 54). Help-seeking is a process of translating the inner world of one’s thoughts and feelings into interpersonal relationships with others (55). Higher levels of perceptual social support from family members and “significant others” predict more positive help-seeking attitudes (56). Individuals with high levels of perceptual social support are more likely to derive positive factors from social support, such as feelings of trust and acceptance, which contribute to the development of positive psychological help-seeking attitudes. People with low levels of appreciative social support are less able to express and disclose to others, have difficulty sharing information about personal distress and, therefore, have negative attitudes toward seeking psychological help (57). The process model of help-seeking defines help-seeking as an interpersonal process in which help-seekers must obtain and access sources of help to realize the process (58). Therefore, in the present study, we hypothesized that help-seeking self-stigmatization further impacted the psychological help-seeking attitude through navigating social support. That is, lower psychological help-seeking self-stigmatization predicts higher PSS in individuals, which results in a more positive psychological help-seeking attitude. Accordingly, Hypothesis #2 could be proposed: appreciative social support mediates the relationship between psychological help-seeking self-stigmatization and the psychological help-seeking attitude.

Optimism is considered to be a generally stable personality trait that reflects the degree to which an individual expects his/her future to develop (59, 60). Optimism is associated with a positive state of mind, perseverance, and effective problem-solving in individuals, as well as good health and even longevity, which are powerful weapons against disease (61). Individuals with high levels of optimism showed more favorable physical (62) and psychological conditions (60), controlling for initial symptom levels. There is an association between MH stigmatization and optimism. Stigmatization influences depression and anxiety by predicting a lower level of optimism (63). Theories on motivational and affective attribution predict that external causal perceptions produce a more positive view of the self relative to internal causal perceptions (64). Individuals with a MH disorder who have high levels of optimism are more likely to adopt external attributions, perceive their MH disorder to be less severe and more manageable and, therefore, exhibit lower self-stigmatization. Moreover, optimism associated with a MH disorder has a “contagious” effect; parents can exhibit optimism about the MH disorder and perceive that the MH disorder is manageable and associated with lower levels of self-stigmatization in their child (65). A reduced level of optimism may influence help-seeking intentions. That is, the less optimistic an individual is, the less likely he/she is to seek help for MH disorder because of a negative attitude toward such help. Research on optimism and coping strategies (66) suggests that optimists tend to adopt reliance on positive, problem-focused coping, are more organized in the face of stressful events, and tend to disengage from goals that are interfered with by the stressor. Those findings further reinforce the idea that optimists are positive copers and are more likely to employ positive coping if suffering from the stress of stigmatization. A high level of optimism promotes help-seeking intentions among adolescents which, in turn, enhances their attitude toward seeking psychological help (67). This finding implies that the self-stigmatization of seeking psychological help may have an impact on the psychological help-seeking attitude through optimism. Accordingly, Hypothesis #3 can be proposed: optimism mediates the relationship between self-stigmatization of psychological help-seeking and the attitude toward seeking psychological help.

**Figure 1:**
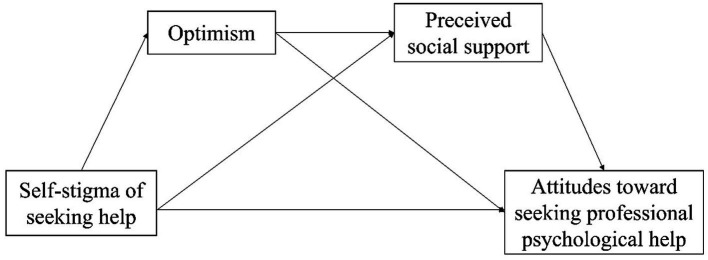
Hypotheses used in this study.

Help-seeking is an important issue for people of all ages, but is particularly important during adolescence because this is a time when lifelong coping patterns can be established (68). However, self-stigmatization can prevent individuals from seeking psychological help and fully engaging with these services (69). Among adolescents, optimism and navigating social support are important prerequisites for mental health (70). They are also significant predictors of psychological resilience and mental health in adolescents (71). Studies have suggested that the ability of an optimist to reduce stress and adjust is mediated by the composition of his/her social networks (72). If faced with a stressful event, optimists tend to hold expectations of a favorable outcome (73), plan for and make the most of their social support (74), and adopt positive coping styles (75). Some surveys on Chinese adolescents have shown that optimism is significantly positively correlated with social support, and the higher the PSS level, the more pronounced is the optimism (76, 77). Optimism can enable adolescents to maintain good and healthy mannerisms to reduce the probability of adverse events. The more optimistic adolescents are, the more social support they can obtain (78), and the better they can perceive social support in the environment. Hence, they can form a positive perception of themselves and their future (79), thereby having a more positive attitude toward seeking psychological help. In summary, Hypothesis #4 can be proposed: PSS and optimism act as chain mediators between self-stigmatization of seeking psychological help and the attitude toward seeking psychological help (Figure 1).

## 2. Materials and methods

### 2.1. Participants

A stratified random sampling method was used to select HSSs from three schools (Chongqing Jiangbei High School, Jiangjin Middle School, Chongqing Qijiang Experimental Middle School) from the main city and districts of Chongqing in China. This method was employed according to the division of administrative regions in Chongqing willing to participate in our study. Written informed consent was obtained from the legal guardian/next of kin of participants, and the content was kept confidential.

### 2.2. Questionnaire

A paper-based questionnaire was distributed to participants who were in years 1–3 of senior high school between 20 February to 20 May 2023. The questionnaire comprised questions on demographic variables (sex, age, family registry, number of siblings, experience of seeking psychological help), Self-Stigmatization of Seeking Help Scale (SSOSH) score, ATSPPH, Perceived Social Support Scale (PASS) score, and Life Orientation Test (LOT) result.

### 2.3. Instruments

#### 2.3.1. Self-stigmatization of seeking help

The SSOSH [developed by Vogel et al. (80) and revised by Zobin and Chuanguo (81)] was used to measure the self-stigmatization attitude. A high total score indicated a high level of self-stigmatization. The scale has 10 items and uses a five-point scale ranging from 1 (“not at all”) to 5 (“fully”). The Cronbach’s alpha for the SSOSH was 0.792.

#### 2.3.2. ATSPPH

ATSPPH [developed by Fischer and Turner (13) and revised by Hao and colleagues (82)] was used to measure the attitude toward seeking psychological help. A high total score indicated a more positive attitude toward seeking PPH. The questionnaire was categorized into four dimensions: self-perception of the need for psychological help; tolerance of stigmatization; interpersonal openness; confidence in MH professionals. The scale has 29 items and is rated on a five-point scale ranging from 1 (“strongly disagree”) to 5 (“strongly agree”). The Cronbach’s alpha for the ATSPPH was 0.866.

#### 2.3.3. PSS

The PASS [developed by Zimet et al. (83) and revised by Jiang (84)] consists of three dimensions: family support, friend support, and other support. The main change is replacement of the words “leaders, relatives, and coworkers” with the words “teachers, classmates, and relatives.” The PASS was used to measure the perception of support from others. A high total score indicated a high perception of social support. Twelve items are used on a seven-point scale ranging from 1 (“strongly disagree”) to 7 (“strongly agree”). The Cronbach’s alpha for the PASS was 0.941.

#### 2.3.4. Optimism

The LOT [developed by Carr (85) and translated by Xue et al. (86)] is used to measure an individual’s tendency toward optimism. A high total score indicates greater optimism. The LOT consists of six items rated on a five-point scale ranging from 0 (“strongly disagree”) to 4 (“strongly agree”). The Cronbach’s alpha for the LOT was 0.662.

### 2.4. Statistical analyses

Statistical analyses were undertaken using SPSS 26.0 (IBM, Armonk, NY, USA). Correlation analyses were conducted to examine bivariate correlations between the main variables. Mediation analyses were undertaken using the “Process” macro of SPSS (model 6). The sample size in the model was set to 5,000. The confidence interval (CI) was set to 95%. An effect was significant if the 95%CI did not contain zero and vice versa. The mediated proportion was calculated by dividing the indirect effect by the total effect. *p* < 0.05 (two-tailed) was considered significant.

Harman’s single-factor test was used to examine the effect of common method bias. Results showed that were 13 with a characteristic root >1. The interpretation rate of the first factor was 22.64%, which was less than the critical standard (40%). Hence, the common method bias of our study was not serious (87).

## 3. Results

### 3.1. Response

A total of 2,276 questionnaires were recovered. A total of 177 invalid questionnaires (e.g., logic inconsistencies and duplicate responses) were excluded. The data from the remaining 2,159 questionnaires (94.86%) were included in the statistical analyses.

### 3.2. Characteristics of study participants

The age range of participants was 14–20 years (16.40 ± 0.78). There were 1,015 (47%) males and 737 (34.1%) were the only child in the family. Also, 1,020 (47.2%) lived in an urban setting, and 212 (9.8%) participants indicated that they had received professional psychological help (Table 1).

**Table 1:** Characteristics of respondents (*n* = 2,159).

Variable	Category	Frequency	Valid percentage (%)
Sex	Men	1,015	47.0
	Women	1,144	53.0
Family registry	Urban	1,020	47.2
	Rural	1,139	52.8
Number of siblings	Only child in the family	737	34.1
	Not only child in the family	1,422	65.9
Experience of psychological help	Yes	212	9.8
	No	1947	90.2

### 3.3. Correlation analysis

Correlation analysis (Table 2) showed that self-stigmatization of seeking psychological help was negatively correlated with ATSPPH (*r* = −0.655, *p* < 0.001), optimism (*r* = −0.524, *p* < 0.001), and understanding social support (*r* = −0.424, *p* < 0.001). Also, ATSPPH was positively correlated with optimism (*r* = 0.471, *p* < 0.001). Comprehension of social support was positively correlated (*r* = 0.398, *p* < 0.001) and optimism was positively correlated with PSS (*r* = 0.448, *p* < 0.001).

**Table 2:** Descriptive statistics and correlations between variables.

	*M*	*SD*	1	2	3	4
1. Self-stigmatization of seeking help	24.41	5.58	1			
2. Attitudes toward seeking professional psychological help	97.68	12.49	−0.655^***^	1		
3. Optimism	21.25	3.67	−0.524^***^	0.471^***^	1	
4. Perceived social support	60.85	13.46	−0.424^***^	0.398^***^	0.448^***^	1

^***^p < 0.001.

### 3.4. Differences based on sex

Differences in self-stigmatization, psychological help-seeking attitudes, optimism, and PSS for seeking psychological help among HSSs of different sexes are shown in Table 3. Specifically, girls had significantly higher levels of psychological help-seeking attitudes (*p* < 0.001), optimism (*p* < 0.001), and comprehending social support (*p* < 0.01) than boys. Boys had significantly higher levels of self-stigmatization for seeking psychological help than girls (*p* < 0.001).

**Table 3:** Differences based on sex.

	Sex	Number	*M* ± SD	*t*
Self-stigmatization of seeking help	Men	1,015	25.25 ± 5.60	6.61^***^
	Women	1,144	23.67 ± 5.46	
Attitudes toward seeking professional psychological help	Men	1,015	94.60 ± 12.55	−11.09^***^
	Women	1,144	100.41 ± 11.79	
Optimism	Men	1,015	20.75 ± 3.70	−6.06^***^
	Women	1,144	21.70 ± 3.59	
Perceived social support	Men	1,015	59.83 ± 14.05	−3.33^**^
	Women	1,144	61.76 ± 12.86	

^**^p < 0.01, ^***^p < 0.001.

### 3.5. Mediation analysis

Self-stigmatization of seeking psychological help (independent variable), attitude toward psychological help (dependent variable), optimism and navigating social support (mediator variables), and sex, being an only child, and experience of psychological help (covariates) were analyzed by regression analysis according to model 6 in the “Process” program. Results (Table 4) showed that self-stigmatization of seeking psychological help significantly negatively predicted a psychological help-seeking attitude (*β* = −1.419, *p* < 0.001), optimism (*β* = −0.336, *p* < 0.001), and PSS (*β* = −0.633, *p* < 0.001). Optimism significantly and positively predicted PSS (*β* = 1.105, *p* < 0.001). Optimism and PSS significantly and positively predicted a psychological help-seeking attitude (*β* = 0.456, *p* < 0.001; *β* = 0.101, *p* < 0.001). With the addition of optimism and PSS as mediating variables, the negative prediction of psychological help-seeking self-stigmatization on a psychological help-seeking attitude remained significant (*β* = −1.165, *p* < 0.001).

**Table 4:** Regression results of chain-mediating effects (*n* = 2,159).

Outcome variable	Predictive variable	*R*	*R^2^*	*F*	*β*	*t*
ATSPPH		0.670	0.449	293.028^***^		
	SSOSH				−1.419	−39.166^***^
	Sex				3.561	8.771^***^
	Number of siblings				−0.087	−0.197
	Experience of psychological help				0.286	0.423
Optimism		0.536	0.287	144.3^***^		
	SSOSH				−0.336	−27.738^***^
	Sex				0.449	3.310^**^
	Number of siblings				−0.008	−0.054
	Experience of psychological help				1.1269	4.979^***^
PSS		0.510	0.259	107.712^***^		
	SSOSH				−0.633	−11.975^***^
	Optimism				1.105	13.707^***^
	Sex				−0.018	−0.034
	Number of siblings				−1.105	−2.010^*^
	Experience of psychological help				3.267	3.839^**^
ATSPPH		0.692	0.478	246.363^***^		
	SSOSH				−1.165	−27.416^***^
	Optimism				0.456	6.969^***^
	PSS				0.101	6.015^***^
	Sex				3.307	8.342^***^
	Number of siblings				0.029	0.069
	Experience of psychological help				−0.684	−1.028

SSOSH, Self-Stigmatization of Seeking Help; ATSPPH, attitudes toward seeking professional psychological help; PSS, perceived social support. ^*^p < 0.05, ^**^p < 0.01, ^***^p < 0.001.

The bootstrap method was used to repeat the sampling 5,000 times to calculate 95%CIs, respectively (Table 5). Testing of the mediating effect showed that the bootstrap 95%CIs for the indirect effects generated by optimism and understanding social support did not contain a value of 0. These data indicated a significant mediating effect of the two mediating variables between self-stigmatization of seeking psychological help and attitude toward psychological help-seeking. This mediating effect consisted of three indirect effects. The first indirect effect was generated by self-stigmatization of seeking psychological help → optimism → psychological help-seeking attitude. The second indirect effect was generated by self-stigmatization of seeking psychological help → PSS → psychological help-seeking attitude. The third indirect effect was self-stigmatization of seeking psychological help → optimism → PSS → psychological help-seeking attitude.

**Table 5:** Standardized direct and indirect paths (*n* = 2,159).

	Effect	BootSE	BootLLCI	BootULCI	Percentage of effect size (%)
Total effect	−0.634	0.016	−0.666	−0.603	-
Direct effect	−0.520	0.019	−0.5578	−0.483	-
Total indirect effect	−0.114	0.013	−0.142	−0.089	-
SSOSH → optimism → ATSPPH	−0.069	0.012	−0.092	−0.045	10.78
SSOSH → PSS → ATSPPH	−0.029	0.006	−0.041	−0.017	4.51
SSOSH → optimism → PSS → ATSPPH	−0.017	0.004	−0.024	−0.010	2.68

SSOSH, self-stigmatization of seeking help; ATSPPH, attitudes toward seeking professional psychological help; PSS, perceived social support.

**Figure 2:**
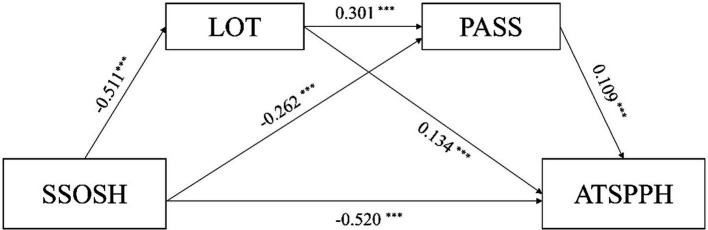
Chain-mediating effect of SSOSH and ATSPPH. SSOSH, self-stigmatization of seeking help; ATSPPH, attitudes toward seeking professional psychological help; PAS, perceived social support. ^***^p < 0.001.

Subsequently, we investigated if there was a significant difference in the indirect effects between different paths. We discovered that indirect effect 1 (self-stigmatization of seeking psychological help → optimism → psychological help-seeking attitude) was significantly higher than indirect effect 2 (self-stigmatization of seeking psychological help → PSS → psychological help-seeking attitude.) and indirect effect 3 (self-stigmatization of seeking psychological help → optimism → PSS → psychological help-seeking attitude.). Also, indirect effect 2 was significantly higher than indirect effect 3 (Figure 2).

## 4. Discussion

The present study suggests that the mediating roles of optimism and PSS contribute to understanding the relationship between self-stigmatization of seeking psychological help and the attitude toward psychological help-seeking in a cohort of Chinese adolescents. Our study elicited five main findings.

First, the results of the present study showed that there were gender differences in self-stigmatization of psychological help-seeking, PSS, and attitudes toward psychological help-seeking, which is consistent with previous studies (88–90). Social culture assigns men the roles of being strong and resilient, and having a mental concerns is seen as a sign of weakness, resulting in men experiencing more severe self-stigma when it comes to seeking psychological help compared to women (91). In addition, women are more empathetic and interpersonally sensitive than men, more interested in establishing a harmonious interpersonal atmosphere, and more likely to perceive social support (92). When facing stressful events, women are better able to mobilize social support resources, and the support from family and friends encourages them to hold a more positive attitude toward seeking psychological help (38). In contrast, social expectations of masculinity require men to be strong and not show weakness, which is characterized by suffering, self-reliance, and reluctance to seek help, which leads to more negative attitudes toward psychological help-seeking among men (93).

Second, consistent with previous research (14), we found that self-stigmatization of seeking psychological help was a significant predictor of a psychological help-seeking attitude, which supports Hypothesis #1. This finding implies that adolescents who hold a deeper self-stigmatization toward seeking PPH tend to have a more negative psychological help-seeking attitude. On the one hand, labeling theory proposes that external stigmatization negatively affects self-stigmatization if individuals are labeled by themselves or others as having MH disorder. Some traditional medical modeling terms and concepts add stigmatization. For example, the more subtle term “disability” has a more nuanced meaning than “disorder” because it encompasses personal characteristics as well as social or environmental barriers, and is less stigmatizing to vulnerable populations (94). The traditionally defined concept of stigmatization associated with labeling can cause individuals to experience shame and trigger negative emotions (95). Many individuals choose not to seek mental-health services because they do not want to be labeled as “mentally ill” or suffer the prejudice and discrimination that comes with that label (96). On the other hand, a psychological help-seeking attitude is closely related to perceptions of mental concerns, understanding and acceptance of psychotherapy and counseling, and trust in counselors (12, 97). An individual may have prejudices about mental concerns or counseling, such as believing that people with “mental disorder” are “useless” and “will be looked down upon by others,” and show a sense of stigmatization and mistrust of psychological help. If this occurs, then the likelihood of seeking PPH when suffering from a mental concerns will be very low.

Third, out findings support Hypothesis #2 that optimism (as an underlying factor) may (at least in part) explain how self-stigmatization of psychological help-seeking is related to the attitude toward psychological help-seeking. Research has shown that as optimism increases, psychological stigmatization associated with an individual’s MH decreases (98), implying that optimism can “buffer” the adverse effects of stigmatization (99). Compared with pessimists, optimists have a greater sense of wellbeing and favor positive interpretations of events. Optimists often use a problem-oriented coping model to deal with real-world problems (100) and are able to flexibly choose adjustment strategies based on their own needs and external resources (101). Optimists are more adept at proactively enhancing and protecting their health (60), demonstrating adaptive coping and strong self-regulation, which may explain why optimists tend to hold a positive attitude toward psychological help-seeking (102, 103).

Fourth, we found that PSS mediated (at least in part) the relationship between self-stigmatization of seeking PPH and a psychological help-seeking attitude, which supports Hypothesis #3. Consistent with the findings of previous studies, PSS had a positive predictive effect on psychological help-seeking behaviors, as evidenced by the fact that a higher PSS level promotes a psychological help-seeking behavior (104–106). Social relationships with others are a basic human need, and are important for healthy adolescent development because early relational experiences influence and shape the quality and expectations of later social relationships (107). Social support denotes the availability of people who make a person feel cared for, valued, loved, and to gain emotional closeness (108, 109). In other words, social support contributes to the enhancement of an individual’s self-worth by providing him/her with the resources to cope with a traumatic event, thereby reducing the negative impacts caused by stressful events (110). Kurzban (111) noted that, even though stigmatization is a behavior that excludes people with certain characteristics from a group, PSS emphasizes the level of support from the community that individuals experience subjectively. The indirect predictive role of self-stigmatization of seeking PPH on ATSPPH may lie in adolescents’ ability to actively utilize their social support strengths to contribute to formation of a positive psychological help-seeking attitude through positive factors, such as the sense of trust and inclusiveness gained from social support. Studies have shown that Chinese people rely heavily on their social networks in coping with psychological distress (112, 113), and that friends and family members play an important part in the help-seeking process. Social networks provide moral support to young people in distress, but also provide them with practical support and encouragement to seek professional help (114).

Fifth, we found that optimism and PSS had a serial mediating role in the relationship between self-stigmatization and ATSPPH, which supports Hypothesis #4. According to field dynamics theory (115), the interplay between proximity dynamics and avoidance dynamics determines an individual’s attitude toward situations. Approach factors positively influence a help-seeking attitude (116, 117). Avoidance factors negatively influence a help-seeking attitude (118, 119). Research has shown that individuals with a higher level of optimism and appreciative social support exhibit a more positive attitude toward psychological help-seeking (67, 120). Thus, optimism and appreciative social support may be protective factors between the stigmatization of psychological help-seeking and ATSPPH. On the one hand, individuals with low self-stigmatization of seeking PPH tend to be associated with a higher level of optimism and PSS, and have more positive ATSPPH by displaying higher optimism in their lives as well as having more social support and believing that they can receive help from others. On the other hand, individuals with a high level of optimism and PSS can integrate their social resources and have an openness to outside help, thus reducing the negative impact of the stigmatization of psychological help-seeking on ATSPPH.

## 5. Limitations and prospects

The present study had three main limitations. First, even though our findings suggest the importance of optimism and navigating social support in the attitude toward psychological help-seeking in adolescents, they do not take into account how these factors change over time. The positive or negative attitudes of an individual toward seeking PPH are not static, but instead change in nature and intensity (106). In the future, longitudinal studies can be used to explore how different factors affect an individual’s attitude toward psychological help-seeking in different time dimensions.

Second, Chinese culture emphasizes emotional restraint and the idea of “saving face” (113), which may view seeking PPH as a sign of weakness. This potential shame may reduce the motivation of Chinese adolescents to seek psychological help (114). In traditional Chinese culture, a physical disorder is more likely to be sympathized with and accepted. For a mental disorder, Chinese people are accustomed to associating it with personal moral character and are more likely to define it as “abnormal,” “defective,” and posing a security risk to themselves and others. Thus, people with a mental disorder in China are more likely to suffer from the stigmatization of a mental disorder (121). Chinese people have a clear family orientation and value personal responsibility and obligation to the family (122). They believe that suffering from a “mental disorder” will increase the burden on the family, which will lead to the impairment of personal value and, therefore, show stigmatization of themselves (123). Influenced by traditional Chinese culture, people are accustomed to associating a mental disorder with a person’s poor moral character, or viewing a mental disorder as “karma.” Once labeled as such, one is ridiculed and belittled, or completely denied the value of one’s existence. Therefore, if suffering from a mental disorder, they are more likely to avoid seeking help to avoid discrimination against themselves and their families (124). In addition, people’s attitudes toward people with various mental health concerns or conditions are not consistent. For example, in some areas of China, some people with hysterical attachment disorder are revered as “daisies.” Also, a disorder experienced by celebrities and media publicity have led to depression being regarded as an “occupational disease,” which is treated sympathetically and even viewed as a manifestation of one’s identity (125). The unique cultural context of China may affect the validity and stability of these findings across cultures. Third, help-seeking attitudes are not equivalent to help-seeking behaviors, and changes in individual intentions and attitudes do not necessarily translate into actual actions (126). Therefore, future research should consider incorporating measures of help-seeking behavior.

## Data availability statement

The raw data supporting the conclusions of this article will be made available by the authors, without undue reservation.

## Ethics statement

The studies involving humans were approved by the Local Research Ethics Committee of Chongqing Normal University. The studies were conducted in accordance with the local legislation and institutional requirements. Written informed consent for participation in this study was provided by the participants’ legal guardians/next of kin.

## Author contributions

NB: Writing – original draft. ZuL: Writing – review & editing. JJ: Data curation. XC: Investigation. ZiL: Investigation. YX: Writing – original draft. XW: Methodology. TZ: Methodology.
